# Comparative single-cell genomics of Atribacterota JS1 in the Japan Trench hadal sedimentary biosphere

**DOI:** 10.1128/msphere.00337-23

**Published:** 2024-01-03

**Authors:** Kana Jitsuno, Tatsuhiko Hoshino, Yohei Nishikawa, Masato Kogawa, Katsuhiko Mineta, Michael Strasser, Ken Ikehara, Jeremy Everest, Lena Maeda, Fumio Inagaki, Haruko Takeyama, IODP Expedition 386 Scientists, Piero Bellanova

**Affiliations:** 1 Graduate School of Advanced Science and Engineering, Waseda University, Shinjuku-ku, Tokyo, Japan; 2 CBBD-OIL, AIST-Waseda University, Shinjuku-ku, Tokyo, Japan; 3 Kochi Institute for Core Sample Research, Japan Agency for Marine-Earth Science and Technology (JAMSTEC), Nankoku, Kochi, Japan; 4 Research organization for Nano and Life Innovation, Waseda University, Shinjuku-ku, Tokyo, Japan; 5 Marine Open Innovation Institute, Shizuoka, Japan; 6 Department of Geology, University of Innsbruck, Innsbruck, Austria; 7 Research Institute of Geology and Geoinformation, AIST Geological Survey of Japan, Tsukuba, Japan; 8 British Geological Survey, Edinburgh, United Kingdom; 9 Advanced Institute for Marine Ecosystem Change (WPI-AIMEC), JAMSTEC, Yokohama, Japan; 10 Department of Earth Sciences, Graduate School of Science, Tohoku University, Sendai, Japan; 11 Institute for Advanced Research of Biosystem Dynamics, Waseda Research Institute for Science and Engineering, Waseda University, Tokyo, Japan; Third Institute of Oceanography Ministry of Natural Resources, Xiamen, China

**Keywords:** Atribacterota JS1, single-cell genomics, Japan Trench, hadal biosphere, carbon cycling, heterotrophy, IODP

## Abstract

Deep-sea and subseafloor sedimentary environments host heterotrophic microbial communities that contribute to Earth’s carbon cycling. However, the potential metabolic functions of individual microorganisms and their biogeographical distributions in hadal ocean sediments remain largely unexplored. In this study, we conducted single-cell genome sequencing on sediment samples collected from six sites (7,445–8,023 m water depth) along an approximately 500 km transect of the Japan Trench during the International Ocean Discovery Program Expedition 386. A total of 1,886 single-cell amplified genomes (SAGs) were obtained, offering comprehensive genetic insights into sedimentary microbial communities in surface sediments (<1 m depth) above the sulfate-methane transition zone along the Japan Trench. Our genome data set included 269 SAGs from Atribacterota JS1, the predominant bacterial clade in these hadal environments. Phylogenetic analysis classified SAGs into nine distinct phylotypes, whereas metagenome-assembled genomes were categorized into only two phylotypes, advancing JS1 diversity coverage through a single cell-based approach. Comparative genomic analysis of JS1 lineages from different habitats revealed frequent detection of genes related to organic carbon utilization, such as extracellular enzymes like clostripain and α-amylase, and ABC transporters of oligopeptide from Japan Trench members. Furthermore, specific JS1 phylotypes exhibited a strong correlation with *in situ* methane concentrations and contained genes involved in glycine betaine metabolism. These findings suggest that the phylogenomically diverse and novel Atribacterota JS1 is widely distributed in Japan Trench sediment, playing crucial roles in carbon cycling within the hadal sedimentary biosphere.

**IMPORTANCE:**

The Japan Trench represents tectonically active hadal environments associated with Pacific plate subduction beneath the northeastern Japan arc. This study, for the first time, documented a large-scale single-cell and metagenomic survey along an approximately 500 km transect of the Japan Trench, obtaining high-quality genomic information on hadal sedimentary microbial communities. Single-cell genomics revealed the predominance of diverse JS1 lineages not recoverable through conventional metagenomic binning. Their metabolic potential includes genes related to the degradation of organic matter, which contributes to methanogenesis in the deeper layers. Our findings enhance understanding of sedimentary microbial communities at water depths exceeding 7,000 m and provide new insights into the ecological role of biogeochemical carbon cycling in the hadal sedimentary biosphere.

## INTRODUCTION

Photosynthetically produced organic matter in shallow seawater is transported to the deep sea and subsequently deposited beneath the seabed (1). As intense degradation of organic matter occurs during sinking, recalcitrant organic matter accumulates on the seafloor and is deeply buried in the underlying sediment (2). Throughout these depositional processes, diverse heterotrophic microorganisms inhabiting the interface between the biologically active surface and energy-limited subseafloor play essential roles in degrading consumable organic matter through aerobic and anaerobic metabolic activities (3, 4).

Hadal oceanic trenches represent the deepest zones of the ocean, ranging from water depths of approximately 6,000–10,916 m, where one oceanic plate is forced to subduct beneath another (5). The V-shaped topography of the trench creates a funneling effect that enhances the accumulation of particulate and dissolved organic matter, characterized by a terminal sink for total organic carbon (TOC). Previous geochemical studies reported unexpectedly high microbial carbon turnover rates in hadal sediments (6), comparable to those occurring in shallower and more productive oceanic regions. This high turnover may be attributed to the substantial input of reactive material transported from the overlying euphotic zone and/or by mass wasting of slope sediments. In the Japan Trench, very high sedimentation rates of diatomaceous hemipelagic mud have been observed (1–3 m/ky) (7). The trench is situated in an area of high oceanic productivity where the cold Oyashio Current interacts with the warm Kuroshio Current (8). Earthquake-related events in the Japan Trench have also been reported to remobilize fine-grained, young TOC-rich sediments over an extensive area of the Japan Trench axes [e.g., the 2011 Tohoku-oki earthquake (Mw 9.1) delivered >1 Tg of C to the Japan Trench] (9). Earthquake-enhanced dissolved organic and inorganic carbon have also been observed in subseafloor sediments (10), potentially sustaining the heterotrophic deep sedimentary biosphere.

The Japan Trench sediments host distinctive microbial communities, different from those found in other hadal trenches. Specifically, in organic-rich surface sediments [approximately 30 cm below seafloor (cmbsf)], heterotrophic microorganisms, such as Bacteroidetes, predominates, whereas more oligotrophic trenches, such as the Mariana and Yap Trenches, exhibit archaeal members within Thaumarchaeota (11, 12). Notably, Japan Trench sediments display a relatively shallow depth of oxygen penetration and an elevated sulfate-methane transition zone (SMTZ) due to heightened methane flux (10, 13). Consequently, anaerobic heterotrophic and methanogenic metabolism plays vital roles in breaking down refractory organic matter in deeper layers (14)

Atribacterota, a gram-negative bacterial phylum, has been identified as the predominant microbial taxon in both organic-rich and anoxic sediments (15), including gas hydrates and methanogenic sediments (16–19). Atribacterota includes the OP9 and JS1 classes, whose members were initially discovered in a yellowstone hot spring and deep marine sediments of the Japan Sea, respectively (20, 21). Previous culture-independent analyses have revealed conserved genomic features among Atribacterota members, including sugar ABC transporters, fermentation pathways of organic acids, and bacterial microcompartment (BMC) gene clusters associated with sugar and aldehyde metabolism (22–24). The novel isolate RT761, *Atribacter laminatus* belonging to the class OP9, exhibits a unique intracytoplasmic membrane surrounding the nucleoid and syntrophic interactions with methanogenic archaea (25). Although no representative of JS1 class has been cultivated, single-cell amplified genomes (SAGs) of JS1 bacteria have been retrieved from various natural environments, including Sakinaw Lake, Canada (26); Etoliko Lagoon, Greece (26); Aarhus Bay, Denmark (22); and marine sediments from the Ross Sea (27). The metagenome-assembled genome (MAG) of the JS1 group in methane-hydrate-bearing sediments revealed the presence of genes encoding osmolytes, suggesting their adaptations to high pressures (18) and survival in the deep biosphere (14). Furthermore, a recent global-scale study on microbial diversity in marine sediments revealed that Atribacterota JS1 relatives are infrequently detected in aerobic sediments of open ocean gyres but are predominant in organic-rich anaerobic sediments along coastal areas (15). This biogeographical distribution underscores the critical ecological role of Atribacterota JS1 as a microbial lineage in global heterotrophic anaerobic microbial ecosystems beneath the seafloor. Nevertheless, the scarcity of genomic data on Atribacterota members from hadal trenches hinders a comprehensive understanding of their phylogenetic characteristics and metabolic functions, critical for their survival in the hadal sedimentary biosphere.

In this study, we employed large-scale SAGs on microbial communities obtained from sediment cores collected at hadal sites in the Japan Trench during the International Ocean Discovery Program (IODP) Expedition 386 (28). The random acquisition of SAGs allows for insights into the structure and function of microbial communities directly sampled from these least-explored natural habitats and reveals the population heterogeneity of the predominant community members in the ecosystem (29, 30). Utilizing microfluidic gel bead-based single-cell genome amplification onboard, we successfully acquired a large number of SAGs attributed to Atribacterota JS1, recognized as the most prevalent heterotrophic bacteria in Japan Trench sediments. A comparative analysis of the metabolic potential, as deduced from the genome data set, verified that certain novel Atribacterota JS1 clades from the Japan Trench actively contribute to the degradation of organic matter in the heterotrophic sedimentary microbial ecosystem at considerable water depths.

## RESULTS

### Hadal sediment samples from the Japan Trench

**Fig 1:**
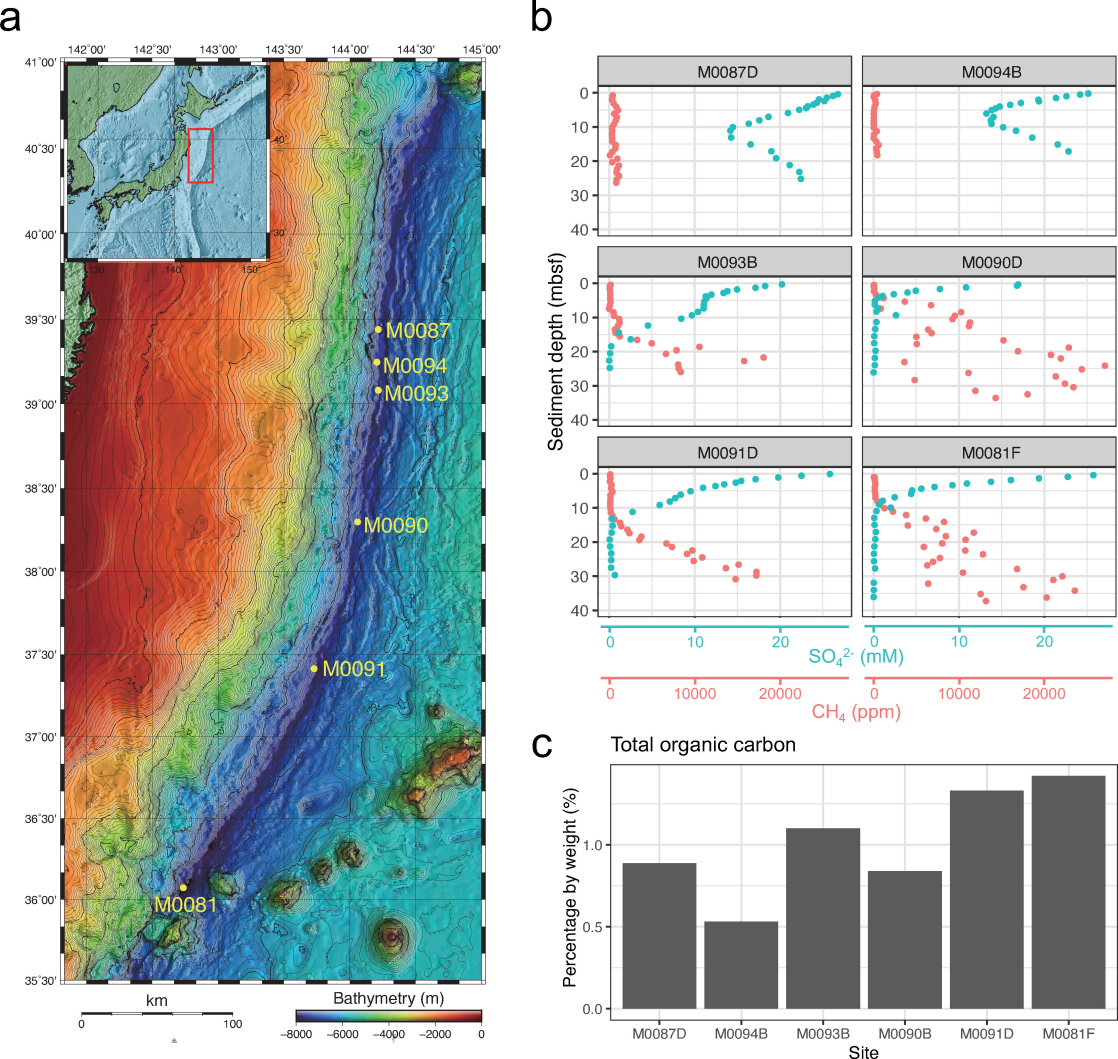
Sampling sites (7,445–8,023 m in water depth) along an approximately 500 km-transect of the Japan Trench during IODP Expedition 386. (a) Map of the Japan Trench region with gravity coring sites M0087, M0094, M0093, M0090, M0091, and M0081. (b) Methane and sulfate concentration profiles of a 40 m deep sediment column. (c) The bar plot shows the concentration of total organic carbon at the bottom end of core section #1 (the uppermost core section after a long GPC was divided into approximately 1-m intervals), with a depth ranging from 10 to 93 cmbsf (Table S1). Geochemical data were obtained according to the IODP Expedition 386 Preliminary Report (32).

Sediment samples were collected from six sites (M0081, M0087, M0090, M0091, M0093, and M0094) along the Japan Trench [7,349–8,020 m below the seafloor (mbsf); Fig. 1a; Table S1] during the IODP Expedition 386 using a Giant Piston Coring system (GPC) of the research vessel (R/V) *Kaimei* in 2021 (Fig. S1). Holes M0081F, M0087D, M0090D, M0091D, M0093D, M0093B, and M0094B were selected to represent the methane and sulfate profiles of the corresponding sites. Onboard geochemical measurements of the pore water and headspace gas samples revealed variations in methane and sulfate profiles among the sites (Fig. 1b). No SMTZ was observed in the sediment core samples at sites M0087D and M0094B, and the low methane concentrations allowed sulfate to diffuse to greater depths. In contrast, the SMTZ was visible in the southern (M0081F and M0091D) and central sites (M0090D and M0093B). The depth of SMTZ at site M0090D was relatively shallower than that of other sites, at around 4.5 mbsf, suggesting relatively high activities of organic matter-fueled sulfate reduction and methanogenesis. The difference in methane profiles among sites may be due to the pervasive existence of earthquake-generated turbidite deposits along the Japan Trench (9, 31), which also affect TOC concentration and microbial fermentation *in situ* (10).

The measured TOC concentrations in the Japan Trench sediment samples ranged from 0.53 to 1.4 wt% (Fig. 1c; Table S2), slightly higher than those of the Ogasawara Trench (0.12–1.57 wt%) and the Mariana Trench (0.16–0.59 wt%) (11). This trend likely stems from the GPC sites of the Japan Trench being situated in a eutrophic ocean with relatively high primary production (33). The TOC concentrations at the southern sites M0081F and M0091D were 1.42 and 1.33 wt%, approximately two times higher than those at the northern sites M0087D and M0094B (0.88 and 0.53 wt%), respectively.

### Taxonomic diversity of microbial communities in the Japan Trench sediments analyzed using amplicon, shotgun metagenomic, and single-cell genome sequencing

**Fig 2:**
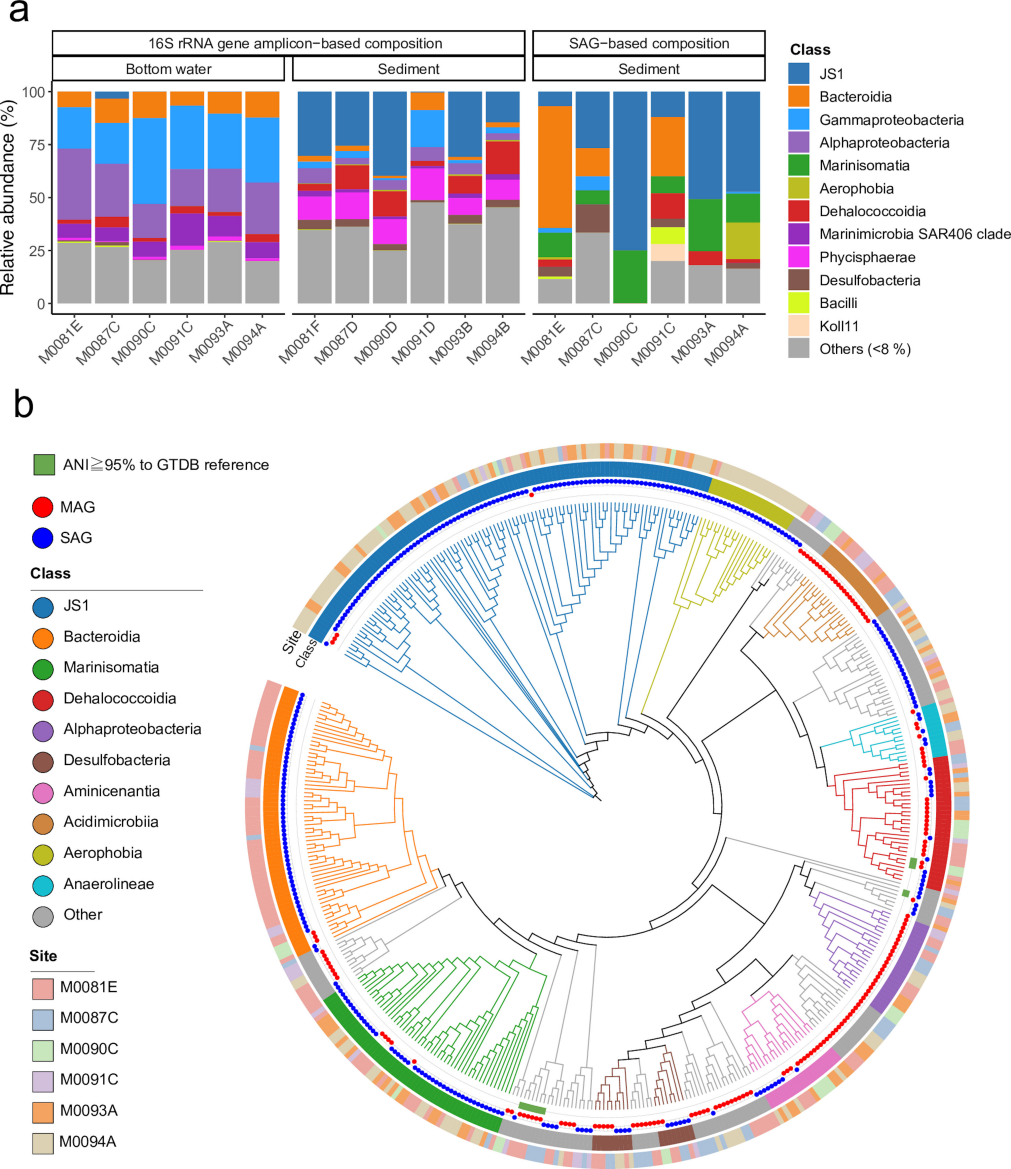
Taxonomic diversity of microbial communities in the Japan Trench sediments analyzed using amplicon, shotgun metagenomic, and single-cell genome sequencing. (a) Microbial community structure of the sediments and bottom water. 16S rRNA gene amplicon-based and SAG-based composition at the class level. Classes with low relative abundances (<8%) are merged as “Others.” (b) Phylogenetic maximum likelihood tree of reconstructed SAGs and MAGs (≥50% completeness and ≥10% contamination) acquired in this study based on the concatenated alignment of 120 bacterial markers in GDTBtk 1.5.0. with FastTree v2.1.10 under WGA (Whelan & Goldman) model. Inner plot: taxonomic novelty. SAGs with ≥95% average nucleotide identity (ANI) to the Genome Taxonomy Database (GTDB) references are indicated by green square plots, indicating that they are the same species as the reference genomes; outer plot: MAG (red) or SAG (blue); inner color bar: taxonomic annotation by class. Minor classes (below the top 10) are merged as “Others”; outer color bar: sediment sampling sites. Tree edges are colored by class.

Sediment samples for DNA analysis were collected at depths ranging from 22 to 108 cmbsf (Table S1). The 16S rRNA gene amplicon-based community composition confirmed the discrete separation of microbial communities between the bottom water and underlying sediments (Fig. 2a; Fig. S2). Bacterial members of the classes Gammaproteobacteria, Alphaproteobacteria, and Marinimicrobia accounted for 43%–62% of the total amplicon sequences from the bottom seawater samples, whereas JS1, Phycisphaerae, and Dehalococcoidia sequences accounted for 16%–61% of the total amplicon sequences from the sediment samples. A sediment sample collected from 22–25 cmbsf at site M0091D had a bacterial community composition similar to that of the bottom water, potentially due to the mixing of seawater and core-top sediments under negative pressure during the GPC drawdown operation. Except for this sample, the sediment samples used in this study were routinely obtained, and the effects of physical disturbances causing seawater mixing were negligible.

A total of 1,886 SAGs were obtained from 187 Gbp of sequencing reads, 306 of which exceeded the thresholds to be defined as medium quality (MQ) and were further investigated in this study. In contrast, 167 MAGs above the MQ were obtained from 32 Gbp sequencing reads. The taxonomic classification of SAGs and MAGs using the Genome Taxonomy Database (GTDB) showed that 306 bacterial SAGs were affiliated with 31 bacterial classes, whereas 167 bacterial MAGs were affiliated with 24 classes (Fig. 2b; Table S3). Most acquired SAGs and MAGs were not taxonomically assigned to any existing GTDB reference classification, potentially representing a new species. Members of JS1, Bacteroidia, Marinisomatia, and Aerophobia were mainly acquired from the SAG data set, whereas Alphaproteobacteria and Acidimicrobia were predominant in the MAG data set. Notably, bacterial lineages detected only through MAG tended to have a higher guanine-cytosine (GC) content (average of 61%). Although the concentration of amplified DNA was sufficient in the single-cell library preparation step, GC-rich templates of the bacterial genome were less amplified by the multiple displacement amplification reaction, resulting in the generation of low-quality SAGs (34). Nevertheless, major bacterial classes in the 16S rRNA gene amplicon-based community composition, including JS1, Bacteroidia, Gammaproteobacteria, Dehalococcoidia, and Desulfobacteria, were also detected in the SAG-based community composition, suggesting that a single cell-based approach has the potential to characterize the microbial communities of dominant populations (Fig. 2a).

Although we obtained numerous qualified SAGs, only 855 (31%) of the gel beads sorted using fluorescence-activated cell sorting (FACS) resulted in 0% genome completeness. Taxonomic classification of contigs within these gel beads showed that 88% of the contigs were primarily derived from bacteria, confirming the presence of highly fragmented bacterial DNA in marine sediments (35). In addition, a comparison of genome quality recovered from fresh and frozen sediments at sites M0087C and M0090C demonstrated an increase in the number of gel beads with 0% genome completeness after storage, indicating that cell lysis and DNA damage likely occurred during the freeze-thaw process (Table S3). This finding underscores the significance of processing fresh samples onboard in real time.

### Genome characteristics and phylogenetic diversity of Atribacterota JS1

A total of 269 JS1 SAGs were obtained from sediment samples collected in the Japan Trench. Among these, 99 (37%) were classified as MQ or high quality. In contrast, from shotgun metagenomic reads, 24 JS1 metagenomic bins were constructed, with only four MAGs (17%) classified as MQ due to a substantial number of contaminated metagenomic bins after *de novo* assembly (58%; Table S4). The average N50 (contigs) was 74 for single-cell genomes and 306 for metagenomic bins, suggesting a higher degree of contig fragmentation in the metagenomic bins (Table S4). As the assembly of multiple species can result in highly fragmented consensus assemblies (36), single-cell sequencing facilitated the recovery of more robust JS1 genomic assemblies compared to shotgun metagenomic sequencing.

Based on the similarity of the single-copy marker genes (≥97%), JS1 clustered into nine clades (clades 1–9; Fig. S3). To improve the quality of the draft genomes, cleaning and co-assembly of a SAG (ccSAG) and composite SAG were performed on SAGs (see Materials and Methods), resulting in 24 genomes with ≥80% completeness and an average estimated genome size of 2.3 Mbp, comparable to that of high-quality JS1 reference genomes (Table S4). However, the number of contigs increased from an average of 699 to 1,624 after co-assembly of the genome pairs of multiple strains. The average nucleotide identity (ANI) values among the acquired clades were <95%, indicating their distinct lineages at the species level. These 24 SAGs (≥80% completeness and ≤10% contamination) and 4 MAGs (≥50% completeness and ≤10% contamination) obtained in this study were used to conduct downstream analyses.

**Fig 3:**
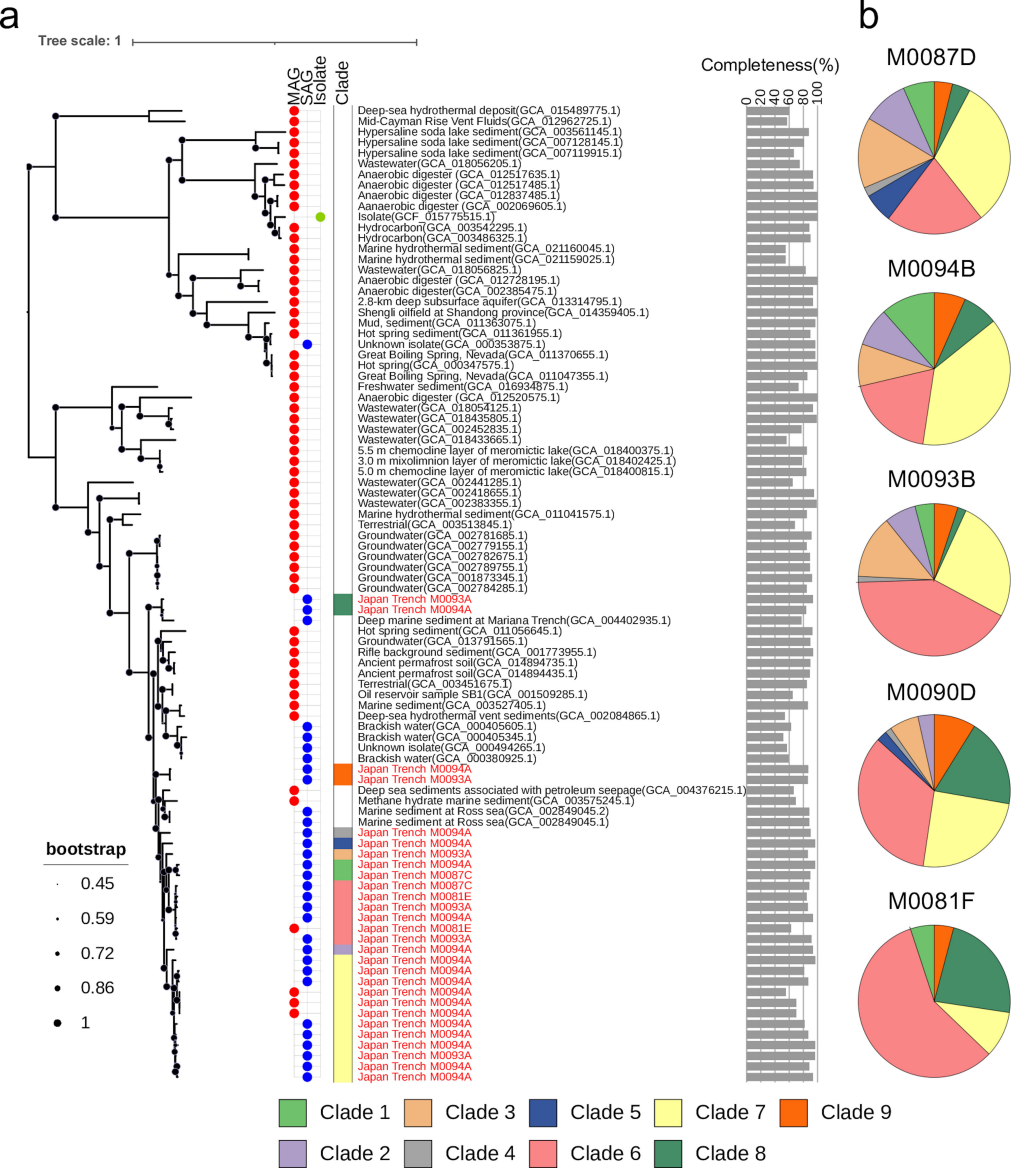
Phylogenetic diversity and geographical distribution of Atribacterota JS1. (a) Phylogenetic maximum likelihood tree of reference SAGs and MAGs (≥50% completeness and ≤10% contamination) of the phylum Atribacterota (OP9 and JS1) and JS1 SAGs (≥80% completeness and ≤10% contamination) and MAGs (≥50% completeness and ≤10% contamination) acquired in this study using the concatenated alignment of 120 bacterial markers in GDTBtk 1.5.0. with FastTree v2.1.10 under WGA (Whelan & Goldman) model. Plot: MAG, SAG, or isolate; Color bar: JS1 clades of the Japan Trench. Text: JS1 in the Japan Trench is in red. Bar plot: genome completeness. (b) Relative abundance of JS1 SAG-affiliated clades along the Japan Trench. JS1 was not detected at site M0091D.

The phylogenetic relationship of our obtained SAGs and MAGs was compared with the genome data set from Atribacterota (OP9 and JS1) in the taxonomic database (≥50% completeness and ≤10% contamination) using concatenated 120 bacterial markers in GDTBtk (Fig. 3a). Therefore, our samples in the tree (clades 1–7 and 9) had less than a 95% similarity to sister clades of taxa reported from the Ross Sea (27), methane hydrate-bearing sediments (ODP Leg 204 Site 1244), and petroleum seepage sites (37). Clade 8 had over a 95% ANI for SAGs from the hadal sediments of the Mariana Trench (accession no. PRJNA526521). Thus, all clades, except clade 8, were distinct from the known taxa in our compiled data set. Furthermore, the phylogenetic analysis of JS1 lineages from the Japan Trench revealed that nine distinct clades were obtained using SAGs, whereas only two were obtained using MAGs (Fig. 3a). These results suggest that the intraspecies diversity of JS1 can be more effectively clarified using a single cell-based approach.

By mapping shotgun metagenomic reads to single-copy marker genes of SAGs with the highest genome completeness within each clade (representative SAGs; Table S4), we determined the biogeographical distribution of SAG-affiliated JS1 clades at six sites along the Japan Trench (Fig. 3b). Notably, two predominant clades (clades 6 and 7) constituted ≥50% of JS1 total abundance, whereas different distribution trends were observed across the north and south. Clade 7 was the most predominant in the northern sites M0087D and M0094B, whereas clade 6 was predominant in the southern sites M0093B, M0090D, and M0081F. No shotgun metagenomic reads obtained from M0091D mapped to any of the JS1 representative SAGs. This result was consistent with the data from the 16S rRNA gene analysis, which showed that the relative abundance of JS1 was extremely low (Fig. 2a). The absence of JS1 at site M0091D may be due to contamination of the seawater during the GPC drawdown operation, drastically altering the microbial composition.

### Fermentation potential

**Fig 4:**
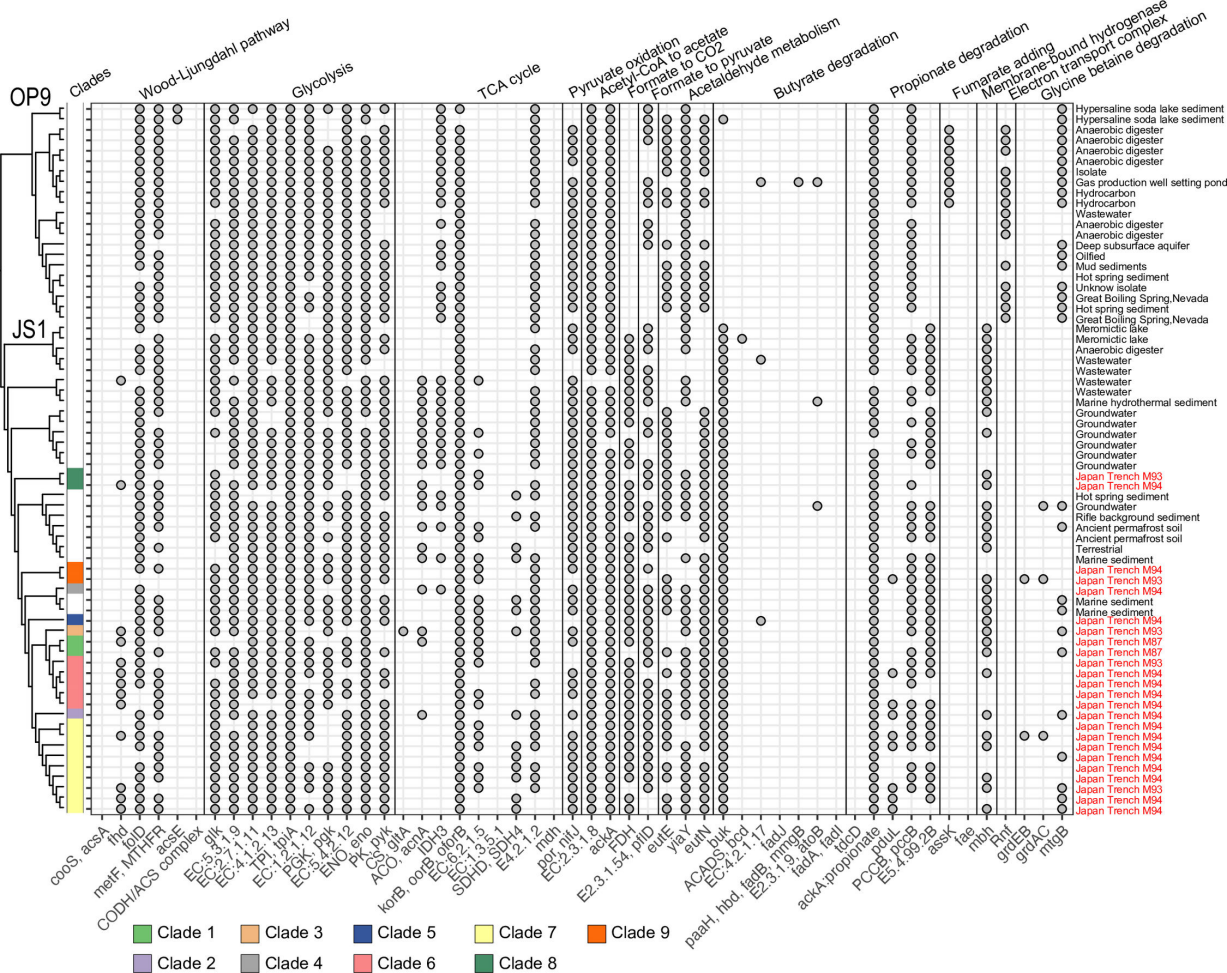
Overview of metabolic functions in Atribacterota (≥80% completeness, ≤10% contamination). y-axis: Atribacterota genomes (upper: OP9, lower: JS1). Isolation sources are indicated in the right column. Colored square indicates clades from Japan Trench sediments, and a blank square indicates Atribacterota genomes from other environments. x-axis: symbol of the key functional genes. Full names and corresponding KO are listed in Table S5.

The high coverage of JS1 SAGs (≥80% completeness and ≤10% contamination) from Atribacterota provided a detailed overview of their physiological potential (Fig. 4; Table S5). Genes associated with glycolysis and pyruvate oxidation were identified as core metabolites in multiple Atribacterota genomes, whereas several genes linked to the tricarboxylic acid (TCA) cycle, such as citrate synthase (CS, *gltA*) and malate dehydrogenase (*mdh*), suggested an anaerobic lifestyle (Fig. 4). Importantly, the genome data set did not contain the complete set of genes encoding the Carbon monoxide dehydrogenase/acetyl-CoA synthase (CODH/ACS) complex, which is essential for the Wood-Ljungdahl pathway. Nevertheless, this lineage may still be capable of catabolizing acetate via the phosphate acetyltransferase-acetate kinase pathway (*pta* and *ackA*). Genes encoding for acetyl-CoA reduction to acetaldehyde (*eutE*) and BMC shell proteins (*eutN*) were detected, supporting the notion of previous studies that Atribacterota employs BMC to store and recycle toxic aldehydes (22, 24).

JS1 members have genes related to the primary fermentation of carbohydrates and the secondary fermentation of organic acids such as propionates. Although propionate kinase (*tdcD*) was not detected, acetate kinase (*ackA*) degrades propionate in the initial step (38). The gene encoding butyrate kinase was the only gene detected in butyrate metabolism. Additionally, formate dehydrogenase (*fdh*) and membrane-bound hydrogenase (*mbh*) were detected in multiple Atribacterota genomes, suggesting a reduction of formate to CO_2_ and hydrogen generation (39).

### Potential of hydrocarbon degradation

Genes involved in anaerobic hydrocarbon degradation have been identified in atribacterial genomes associated with hydrocarbon-enriched environments (40). Therefore, our objective was to pinpoint the genes associated with hydrocarbon degradation in JS1-related SAGs obtained from the Japan Trench. In several OP9 clades, we found a putative AMP-dependent CoA ligase/synthetase (*assk*) crucial for fumarate addition, although it was notably absent in JS1. The presence of fumarate addition enzyme operons was not observed in JS1 genomes from groundwater or brackish water (40), suggesting that anaerobic hydrocarbon degradation is not a prevalent metabolic trait within the JS1 lineages.

### Comparative genomic analysis among habitats

**Fig 5:**
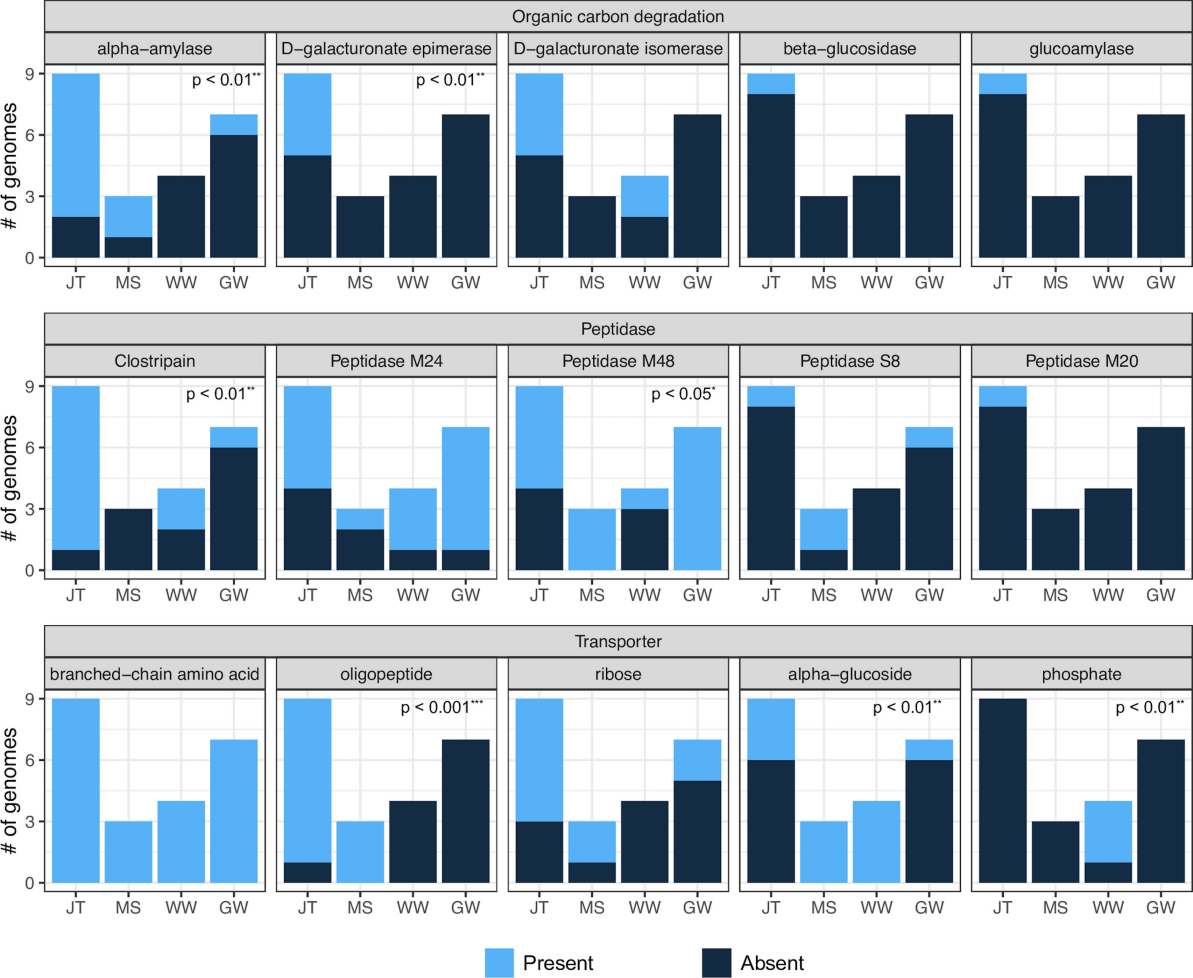
Comparison of organic matter degradation potentials in JS1 species among the Japan Trench (*n* = 9), shallow marine sediment (*n* = 3), wastewater (*n* = 4), and groundwater (*n* = 7). The number of genomes with and without a functional gene (presence, blue; absence, dark blue) was compared using Fisher’s exact test. The *P*-value is indicated in the top right corner of each figure. x-axis: JT, Japan Trench; MS, marine sediment; WW, wastewater; GW, groundwater.

We assessed the metabolic potential for organic carbon degradation among various JS1 lineages. Representative SAGs, with ≥80% completeness and ≤10% contamination, were selected to represent JS1 species from Japan Trench sediments. These were then compared with a reference genome data set, which included data sets from diverse environments, including marine sediments in the western Ross Sea (27), groundwater at the Crystal Geyser (41), and wastewater in a treatment plant (42). The frequencies of genes encoding carbohydrate degradation enzymes, extracellular peptidases, and ABC transporters were compared among JS1 members across different habitats (Fig. 5). Results indicated a frequent detection of the carbohydrate-degrading enzyme α-amylase encoding gene in JS1 genomes from the Japan Trench and marine sediments (Fisher’s exact test; *P* < 0.001**), whereas it was absent in groundwater and wastewater habitats. Moreover, genes encoding D-galacturonate degradation enzymes (epimerase and isomerase) were identified in multiple JS1 genomes from the Japan Trench. D-galacturonate serves as the primary component of pectin in numerous plants (43). Regarding extracellular peptidases, clostripain-encoding genes were more frequently detected in the Japan Trench and wastewater habitats (Fisher’s exact test; *P* < 0.01**) compared to groundwater and marine sediments. For the ABC transporter, the gene encoding the branched-chain amino acid transporter system was present across all habitats, whereas the oligopeptide transporter system was exclusively observed in the Japan Trench and marine sediments (Fisher’s exact test; *P* < 0.001***). In addition, the ribose transporter system was detected in multiple JS1 genomes from the Japan Trench. In contrast, phosphate transporters were only observed in the JS1 genome from wastewater (Fisher’s exact test; *P* < 0.01**), likely due to the generally high phosphate concentration in wastewater (44).

### Correlation between the Japan Trench JS1 and geochemical data

**Fig 6:**
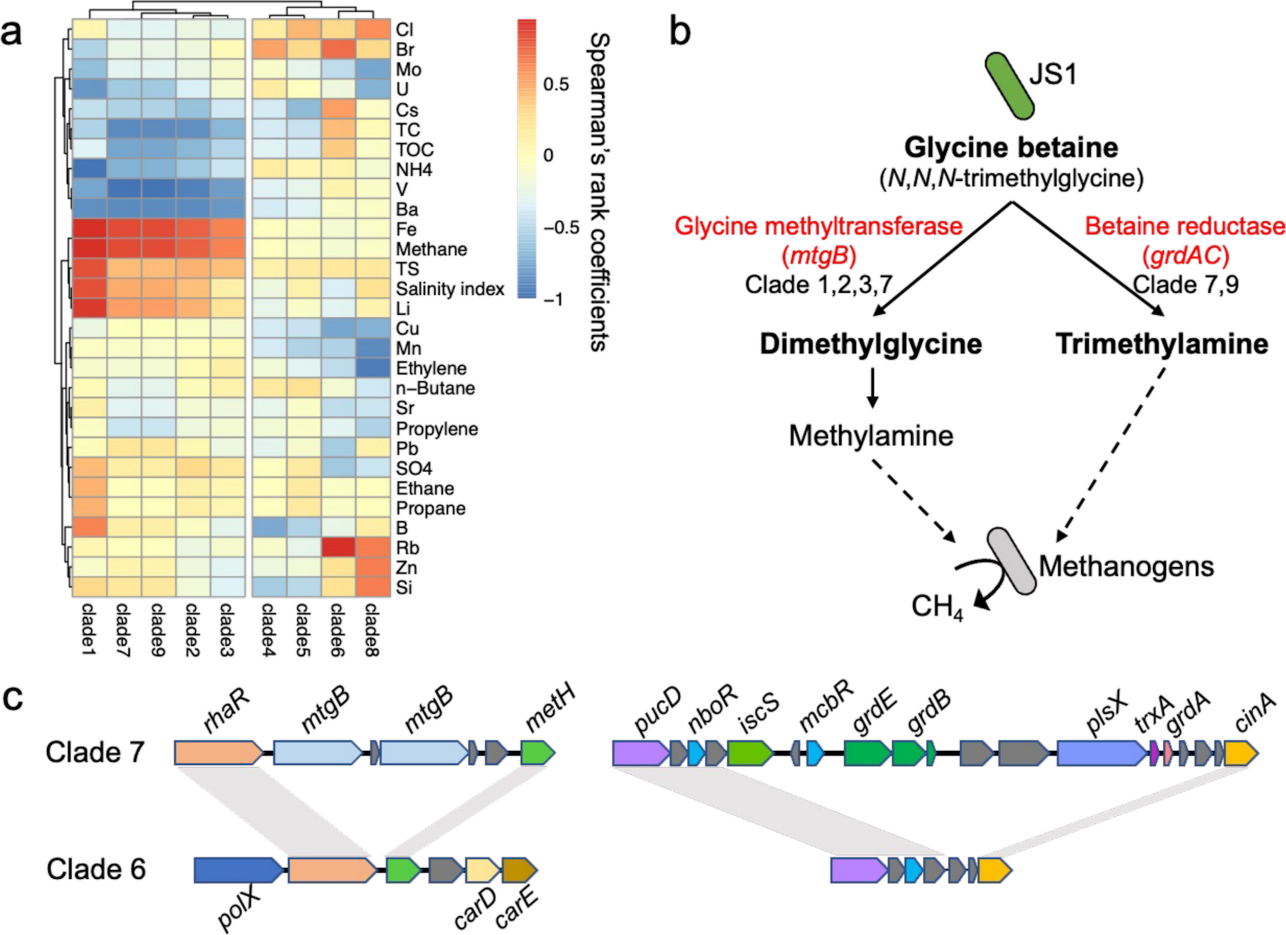
The correlation between the Japan Trench JS1 and geochemical data. (a) The heatmap shows Spearman’s rank correlation coefficient between the abundance of each JS1 clade and geochemistry data. (b) Glycine betaine degradation pathways in particular JS1 clades and possible stimulation of methanogenesis. (c) Synteny analysis of contigs near the glycine betaine corrinoid protein Co-methyltransferase gene (*mtgB*) and betaine reductase genes (*grdA*). Gene color: gene types. Gene label: *rhaR*, HTH-type transcriptional activator; *mtgB*, glycine betaine methyltransferase; *metH*, methionine synthase; *polX*, DNA polymerase/3´−5´ exonuclease; *carD*, caffeyl-CoA reductase-Etf complex subunit; *carE*, caffeyl-CoA reductase-Etf complex subunit; *pucD*, probable xanthine dehydrogenase subunit D; *nboR*, nicotine blue oxidoreductase; *iscS*, Cysteine desulfurase IscS; *mcbR*, HTH-type transcriptional regulator; *grdE*, glycine reductase complex component B subunits alpha and beta; *grdB*, glycine reductase complex component B subunit gamma; *plsX*, phosphate acyltransferase; *trxA*, thioredoxin; *grdA*, glycine/sarcosine/betaine reductase complex component A; *cinA*, putative competence-damage inducible protein.

We conducted a comparative analysis of the metabolic functions among various JS1 clades and considered their correlation with environmental factors in Japan Trench sediments. Spearman’s rank correlation analysis highlighted strong associations between the abundances of JS1 clades 1, 2, 3, 7, and 9 and the *in situ* methane concentration (Fig. 6a; Fig. S4a). Notably, the methane-associated JS1 clades contained genes related to glycine betaine (*N*, *N*, *N*-trimethylglycine) degradation. Specifically, these clades exhibited either the glycine betaine corrinoid protein co-methyltransferase gene (*mtgB*) or glycine/sarcosine/betaine reductase complex genes (*grdAC*), facilitating the conversion of glycine betaine to dimethylglycine or trimethylamine (Fig. 6b), which are essential substrates for methylotrophic methanogenesis (45). The proximity of *grdAC* to the glycine reductase complex (*grdBE*) was also identified (Fig. 6c). In contrast, clades weakly correlated with methane (clades 4, 5, 6, and 8) lacked *mtgB* and *grdAC* genes (Fig. 4). Notably, clade 6 was devoid of genes related to glycine betaine metabolism (Fig. 6c). Furthermore, an investigation into archaeal diversity in the Japan Trench sediments using widely employed 515F/806R primers for microbiome studies (15) revealed that archaeal reads constituted only 8% of the total reads (Fig. S4b). Methylotrophic methanogens, specifically relatives of *Ca*. Methanofastidiosa (46), accounted for 1% of archaeal abundance (Fig. S4c). This suggests that exceedingly rare methylotrophic methanogens may utilize noncompetitive substrates *in situ* rather than relying on a more reductive environment below the SMTZ during the burial process. In summary, the integration of genome-based functional analysis and geochemical data revealed the potential of specific JS1 bacteria to serve as substrates for methanogenesis, contributing to the heterotrophic carbon cycling observed in the sediments of the Japan Trench.

## DISCUSSION

Previous geological studies utilizing hydroacoustic sub-bottom profiles and sediment cores along a transect of the Japan Trench axis have unveiled the extensive influence of event deposits on sedimentary succession (47, 48). In this study area, organic-rich event layers associated with the 2011 Tohoku-oki earthquake were identified in the uppermost sediments at the southern site M0081 but were absent at the northern site M0094 (9). The mean sedimentation rate at the southern sites, 5.44 m/kyr, surpassed those in the central (approximately 2.0 m/kyr) and northern (1.17 m/kyr) Japan Trench (47). Variances in lateral sediment transport systems from the upper slope to the hadal trench may account for the differences in methane and sulfate profiles and TOC observed among the six sites, particularly influencing the quantity of organic matter available for heterotrophic microbial communities in the hadal sedimentary biosphere.

We obtained 306 SAGs above the MQ using the SAG-gel system from hadal sediments at depths exceeding 7,000 m along an approximately 500 km transect of the Japan Trench. To date, single-cell genome sequencing of trench sediments has been performed by a single case study, wherein 12 Parcubacteria-related SAGs with 2%–66% completeness were obtained from a single site in the Mariana Trench (49). In a shallower marine environment, a study that acquired SAGs from an 8 m-deep sediment at the Atlantis Massif yielded 227 SAGs with 0%–29% completeness when cells were randomly sorted using FACS from frozen sediment samples preserved with recommended fixatives (50). In a similar scenario of randomly acquiring SAGs from frozen sediments in the Baltic Sea, 46 SAGs with 1%–75% completeness were obtained (51). In this study, we have notably enriched the genetic information by successfully obtaining 24 JS1 SAGs with ≥80% completeness, spanning nine clades from six different locations in the Japan Trench.

Despite the large number of qualified SAGs obtained in this study, numerous gel beads sorted into 384-well plates showed 0% genome completeness, as assessed using CheckM estimates based on single-copy marker genes. These gel beads predominately contain contigs derived from bacteria, suggesting the presence of abundant fragmented bacterial DNA in marine sediments (35). A comparison of SAG completeness in samples processed from both fresh and frozen sediments revealed that DNA damage due to frozen storage significantly affects genome quality. The utilization of a portable SAG-gel system during the IODP Expedition 386 underscores the importance of conducting single-cell genome amplification from fresh sediment samples immediately after core recovery.

Single-cell genome analysis enabled the identification of highly diversified JS1 (clades 1–9) in the Japan Trench hadal sediments. In contrast, shotgun metagenomic analysis identified only two dominant JS1 clades. Conventional metagenomic approaches often struggle to bin contigs into closely related species (52). However, the single cell-based approach used in this study demonstrated a clearer representation of the interspecies diversity of JS1. Furthermore, the metabolic pathways shared by all JS1 members across various habitats were glycolysis and fermentation. The incomplete TCA cycle in the JS1 lineage, particularly the absence of genes encoding citrate synthase (24), suggests that the fermentation pathway is more advantageous in anoxic marine sediments (14). Although the Wood-Ljungdahl pathway is functional in Atribacterota (24, 53), we could not confirm it due to the absence of a complete set of genes encoding the CODH/ACS complex (Fig. 4). Therefore, we speculate that acetogenesis is unlikely to occur via the Wood-Ljungdahl pathway.

In the comparative genomic analysis of different habitats, JS1 SAGs from the Japan Trench sediments frequently exhibited coding genes associated with the degradation of organic matter, such as α-amylase, D-galacturonate, and clostripain. A prior metagenomic analysis indicated a higher relative abundance of diverse organic matter hydrolysis-encoding genes in organic-rich sediments, suggesting a significant correlation between microbial organic matter degradation potential and TOC content (54). Considering the frequent occurrence of organic carbon export to the Japan Trench (48), active bacterial degradation and the positive uptake of carbon sources from the surrounding environment were inferred. The organic matter in the entire Japan Trench area primarily comprises marine algae and diatoms, potentially reflecting the high levels of primary production supported by nutrient-rich surface waters in these regions (31, 47). Notably, several JS1 species in the Japan Trench demonstrated the potential to degrade D-galacturonate, a major component of terrestrial plants, suggesting their capability to derive energy from both marine and terrestrial organic matter with carbon-rich structural polymers (55). Clostripain, an arginine-specific endopeptidase secreted in archaeal members of the subseafloor, was detected in JS1 SAGs (56). Considering its stability and activity in subseafloor sediments (57), clostripain may contribute to the persistence of Atribacterota in the hadal sedimentary biosphere. Moreover, JS1 members in the Japan Trench frequently harbored genes encoding transporters for ribose, oligopeptide, and branched-chain amino acids, aligning with previous findings indicating their role in transporting amino acids and sugars from the surrounding environment to the cellular interior, where they are metabolized for energy to support growth (19, 22–24, 27). For example, ribose is the most abundant organic carbon in marine sediments, and the ribose bisphosphate pathway yields four ATP molecules per ribose, with end products including CO_2_, H_2_, and acetate (58).

A previous study employing 16S rRNA amplicon analysis demonstrated an association between the abundance of the Atribacterota phylum and methane concentration (59). In this study, single-cell genome analysis enhanced taxonomic resolution for JS1 phylotypes, revealing variations in methane association; those strongly linked to methane exhibited genes related to glycine betaine metabolism (Fig. 6). Glycine betaine, an osmoprotectant found across all three domains of life (60, 61), has been previously observed in elevated concentrations in saline environments (62). Its expression under high-pressure culture conditions may persist in deeper habitats (63, 64). Methylamines derived from glycine betaine serve as noncompetitive substrates for methanogenesis; however, most methanogens are incapable of efficiently converting glycine betaine to methane (45). Therefore, they rely on bacteria to convert glycine betaine to methylamines. Atribacterota JS1, a predominant bacterial phylum in coastal marine sediments, excels in organic matter degradation (19) and could serve as a crucial substrate for methanogenesis during burial. Notably, glycine betaine metabolism was not a conserved feature among JS1 members (Fig. 4), suggesting that environmental heterogeneity may have influenced the natural selection of locally adapted genotypes.

In summary, employing a single cell-based approach, this study demonstrated that significant SAGs encompass more diverse lineages than those previously inferred from conventional shotgun metagenomic approaches. Novel Atribacterota JS1 lineages are widely distributed in the Japan Trench and play a significant ecological role as drivers of biogeochemical carbon cycling in the hadal sedimentary biosphere.

## MATERIALS AND METHODS

### Sampling and handling

We collected hadal sediment samples from six sites along the Japan Trench in the Pacific Ocean during the IODP Expedition 386 (13 April–1 June 2021; Fig. 1; Table. S1). Sediment samples were acquired using a 40 m-GPC system onboard the R/V *Kaimei*. Multiple holes were established at each site (holes A–F) through successive GPC deployments, with each deployment comprising a pilot hole (A, C, and E) and an accompanying GPC hole (B, D, and F) (32). The specific holes selected for single-cell genome, metagenome, and geochemical analyses at each site are summarized in Table S1. Following the retrieval of the GPC assembly on the deck, the GPC core was sectioned at approximately 1-m intervals (Fig. S1). The collected sediment cores were promptly subsampled for geochemical and microbiological analyses. Time-sensitive samples for microbiological DNA analysis and headspace gas analysis were collected near the center of the freshly cut end of each core section using a sterile syringe. For single-cell genome analysis, sediment samples were exclusively collected from the bottom end of core section 1 at a depth of 63.5–97 cmbsf (Fig. S1; Table S1). Sediments samples for single-cell genome analysis were subjected to the SAG-gel procedure (refer to the “single-cell sequencing” section) immediately after core recovery, whereas the remaining sediments were suspended in 20% glycerol Tris-EDTA (TE) buffer (vol/vol) and stored in −80°C (Fig. S1). In addition to sediment cores, bottom water samples were obtained from trigger cores. These bottom water samples were filtered immediately after sampling using 5 µm and 0.22 µm pore-size filters. Both sediment samples and bottom water filters were stored untreated at−80°C.

### Geochemical analysis

All geochemical measurements were conducted in accordance with the IODP Expedition 386 Preliminary Report (32). In brief, interstitial water was extracted at 50-cm intervals using Rhizon samplers (Rhizosphere Research Products, Netherlands). Sulfate concentrations (SO_4_^2-^) were determined using an 882 compact ion chromatograph (Metrohm, Swiss). Methane concentrations were measured following standard procedures for headspace gas sampling and analysis (32). The TOC content of the sediments was measured using a CS744 LECO carbon-sulfur analyzer after treating samples with 12.5% HCl to remove calcium carbonate. All analyses were performed at the MARUM Bremen Core Repository, University of Bremen.

### Single-cell genome sequencing

Microbial suspensions were generated from sediment samples under two different conditions. For sites M0081E, M0087C, M0090C, and M0091C, microbial suspensions were prepared from freshly collected sediments onboard, whereas additional suspensions were prepared from frozen sediments at sites M0087C, M0090C, M0093A, and M0094A. To isolate microbial cells from the sediment under both conditions, 10 mL of phosphate-buffered saline (PBS) was added to approximately 0.7 g of sediment and vortexed vigorously (30 s ON, 30 s OFF × 3). After allowing the sediment to settle at room temperature for 20 min to enable precipitation of the large particles, the supernatant was filtered through a 10 µm filter (Merck, Germany). Subsequently, the supernatant was transferred to a new 15 mL tube (Fastgene, Japan) and centrifuged at 10,000 × *g* for 5 min at room temperature to concentrate the final volume to 100 µL. The cell fraction was quantified with SYBR Green I (×10) and diluted to 4.2 × 10^4^ cells/µL with PBS. The original solution was used if the cell concentration did not meet the required standard values. Single-cell genome sequencing was performed using the SAG-gel (single-cell amplified genomes in gel beads sequencing) platform following the published protocol (29, 65). A microfluidic droplet generator (Dolomite, UK) was utilized onboard immediately after core recovery or −80° storage to capture individual microbial cells in agarose gel beads. Cell lysis and subsequent whole-genome amplification (WGA) were performed using agarose gel electrophoresis, employing this single cell-based molecular ecological approach. After WGA reaction, gel droplets were stored at 4°C in TE buffer until FACS for 3 months. The gel beads were washed and stained with 10× SYBR Green I (Thermo Fisher Scientific). A total of 2,785 fluorescence-positive gel beads were sorted using FACS Melody (Becton Dickinson, USA) into 384-well plates. Only gel beads prepared from freshly collected sediment samples underwent a second round of WGA (29). SAG libraries were prepared and sequenced by bitBiome, Inc. (Tokyo, Japan; https://bitbiome.co.jp).

### DNA sequencing

DNA extraction from 5 g of frozen sediment was performed using the PowerMax Soil DNA Isolation Kit (Qiagen, Germany), and DNA from bottom water filters was extracted using the Denay Plant Mini Kit (Qiagen), following the manufacturer’s instructions. The V4 hypervariable region of the 16S rRNA gene was amplified using PCR with universal primers 515F/806R (15), with an index and adaptor. The 25 µL PCR mixture contained 10 µM of each primer, 2.5 µL of template DNA, 1 × MightyAmp Buffer Ver.3, 0.5 µL of MightyAmp DNA polymerase (TaKaRa Bio, Japan), and 8.5 µL distilled water. PCR commenced with 2 min at 98°C, followed by a maximum of 35 cycles involving denaturation at 98°C for 10 s, annealing at 55°C for 15 s, and elongation at 68°C for 30 s. After electrophoresis, PCR products of the target size were excised from the agarose gel and purified using a PCR and Gel Purification Kit (Takara Bio). Concentrations of purified PCR products were measured, and equal amounts of each sample were mixed to prepare a sequence library. Sequencing was performed using a MiSeq Reagent Kit v3 (600 cycles; Illumina, USA) following the manufacturer’s instructions. Quality control included primer trimming with cutadapt, repair reads with Bbmap, and sequence length screening using FLASH v2.2.00 (66) and Fastp v0.20.1 (67). 16S rRNA gene analysis was performed using QIIME2-2020.11 (68). Distance matrices were generated using the “vegan” package, and ordinations were visually compared through principal coordinate analysis based on the weighted UniFrac distance matrix. Amplicon sequence variants were constructed using DADA2. Taxonomic assignment employed the q2-feature-classifier (69) and classify-sklearn naïve Bayes taxonomy classifier against the Silva 138 99% OTUs full-length sequence. Shotgun metagenome libraries were prepared from 5 ng of extracted DNA using Illumina DNA Prep (Illumina), and sequencing was conducted on an Illumina HiSeq X sequencer (Macrogen, Japan).

### SAG and MAG assemblies and quality control

The single-cell genome sequencing reads were assembled *de novo* using SPAdes v3.14.0 (70) and shotgun metagenomic reads were assembled *de novo* using MetaWRAP v1.3.2 (71) for binning. Subsequently, the assemblies were refined in DAS_tool v1.1.1 (71). The quality assessment of both MAGs and SAGs was performed using CheckM v1.1.3 (72), and the corresponding genome quality is shown in Table S3. The categorization of genome quality adhered to the Minimum Information about a Single Amplified Genome and the Minimum Information about a Metagenome-Assembled Genome standards (73).

### Grouping same strain single-amplified genomes into ccSAG or composite SAG

SAGs with genome completeness ≥50% and contamination ≤10% were selectively chosen using CheckM (72). The ANI for the selected SAGs was computed through FastANI v1.33 (74). The homology of common single-copy marker genes obtained using the CheckM taxonomy workflow was determined using blastn 2.12.0+ with default parameters. SAGs exhibiting single-copy marker gene homology ≥97% were classified within the same clade (Fig. S3). To enhance the coverage of SAGs, ccSAG and composite SAG were performed by merging SAGs from the same site with ANI ≥95% and single-copy marker gene homology ≥99%. ccSAG was performed as previously described (75), and composite SAG was performed using SPAdes (70).

### Gene prediction, functional annotation, and phylogenetic analysis

Coding sequences (CDS), rRNAs, and tRNAs were extracted from all SAGs or MAGs using Prokka 1.14.6 (76). A phylogenetic maximum likelihood genome tree was generated by aligning 120 single-copy proteins from GTDB-Tk 1.5.0 using FastTree v2.1.10 under the WGA (Whelan & Goldman) model (77) integrated into GTDB (78). The resulting phylogenetic tree was visualized using iTOL v6.8.1 (79). Taxonomic novelty was assessed through the classify workflow of GTDB-Tk, where ANI ≥95% indicated the same species cluster. Within this workflow, CDS were assigned to KEGG Orthology (KO) identifiers using eggNOG-mapper v2.1.6 (80). KO for functional genes such as putative hydrocarbon-degrading genes (40) and peptidase-encoding genes (54) has been described previously. The enzymatic function was confirmed if we could detect all enzyme complex genes. The number of JS1 genomes with and without those functional genes was compared using Fisher’s exact test. The gggenomes v0.9.5.9000 package was used to visualize the synteny regions among the genomes.

### Biogeography of JS1 SAG-affiliated clades

Relative abundances of JS1 SAG-affiliated clades were determined based on the mean coverage calculated using CoverM v0.6.1 (https://github.com/wwood/CoverM). The SAG with the highest genome completeness within each clade served as the representative sequence, and single-copy marker genes from CheckM were extracted. The similarity of the conserved region (≥59,163 bp) of single-copy marker genes between different clades was <96%. Subsequently, shotgun metagenomic reads were mapped to conserved single-copy marker genes of representative SAGs using ≥99% identity and ≥150 base pairs alignment length. The following command was employed: coverm genome methods mean–proper-pairs-only–min-read-aligned-length-pair 150–min-read-percent-identity-pair 99.

## Data Availability

Single-cell genome sequencing reads from sediment samples at sites M0081E, M0087C, M0090C, M0091C, M0093A, and M0094A are accessible on the SRA under the accession number PRJNA983928. The shipboard data used in this study are available in the IODP Expedition 386 Preliminary Report (http://publications.iodp.org/proceedings/386/386title.html).

## SUPPLEMENTAL MATERIAL

Supplemental information msphere.00337-23-s0001.docx Supplemental figures and captions for the supplemental tables.

Table S1 msphere.00337-23-s0002.xlsx Sampling locations and holes during IODP Expedition 386.

Table S2 msphere.00337-23-s0003.xlsx Summarized geochemical data.

Table S3 msphere.00337-23-s0004.xlsx The quality of genomes obtained using single-cell and shotgun metagenomic sequencing in this study.

Table S4 msphere.00337-23-s0005.xlsx The genome statistic of JS1 SAGs ({greater than or equal to}80% completeness and {less than or equal to}10% contamination) and MAGs in the Japan Trench ({greater than or equal to}50% completeness and {less than or equal to}10% contamination).

Table S5 msphere.00337-23-s0006.xlsx Detection of functional genes in all Atribacterota genome dataset ({greater than or equal to}80% completeness and {less than or equal to}10% contamination)

ASM does not own the copyrights to Supplemental Material that may be linked to, or accessed through, an article. The authors have granted ASM a non-exclusive, world-wide license to publish the Supplemental Material files. Please contact the corresponding author directly for reuse.
