# Supplementary material for: Comparative single-cell genomics of Atribacterota JS1 in the Japan Trench hadal sedimentary biosphere
Source: mSphere. 2024 Jan 3;9(1):e00337-23. doi: 10.1128/msphere.00337-23 (PMC10826368; doi:10.1128/msphere.00337-23)
Supplement: Supplemental information — Supplemental figures and captions for the supplemental tables. [file msphere.00337-23-s0001.docx]

Supplementary information

**Comparative single-cell genomics of Atribacterota JS1 in the Japan Trench hadal sedimentary biosphere**

Kana Jitsuno^a,b^, Tatsuhiko Hoshino^c^, Yohei Nishikawa^b,d^, Masato Kogawa^d^, Katsuhiko Mineta^b,d,e^, Michael Strasser^f^, Ken Ikehara^g^, Jeremy Everest^h^, Lena Maeda^i^, Fumio Inagaki^d,i,j^#, Haruko Takeyama^a,b,d,k^#, and IODP Expedition 386 Scientists^†^

^a^Graduate School of Advanced Science and Engineering, Waseda University, Shinjuku-ku, Tokyo 169-8555, Japan

^b^CBBD-OIL, AIST-Waseda University, Shinjuku-ku, Tokyo, 169-8555, Japan

^c^Kochi Institute for Core Sample Research, Japan Agency for Marine-Earth Science and Technology (JAMSTEC), Nankoku, Kochi 783-8502, Japan

^d^Research organization for Nano and Life Innovation, Waseda University, Shinjuku-ku, Tokyo, 169-8050, Japan

^e^Marine Open Innovation Institute, Shizuoka, 424-0922, Japan

^f^Department of Geology, University of Innsbruck, Innsbruck, Austria

^g^AIST Geological Survey of Japan, Research Institute of Geology and Geoinformation, Tsukuba 305-8567, Japan

^h^British Geological Survey, Edinburgh EH14 4AP, United Kingdom

^i^Advanced Institute for Marine Ecosystem Change (WPI-AIMEC), JAMSTEC, Yokohama 236-0001, Japan

^j^Department of Earth Sciences, Graduate School of Science, Tohoku University, Sendai 980-8574, Japan

^k^Institute for Advanced Research of Biosystem Dynamics, Waseda Research Institute for Science and Engineering, Waseda University, 169-8050, Japan

^†^A full list of IODP Expedition 386 Scientists is presented at the end of this paper.

**#Address correspondence to:**

Haruko Takeyama, Graduate School of Advanced Science and Engineering, Waseda University, Okubo 3-4-1, Shinjuku-ku, Tokyo 169-8555, Japan. E-mail: haruko-takeyama@waseda.jp

Fumio Inagaki, Advanced Institute for Marine Ecosystem Change (WPI-AIMEC), Japan Agency for Marine-Earth Science and Technology (JAMSTEC), Showa-machi 3173-25, Kanazawa-ku, Yokohama 236-0001, Japan. E-mail: inagaki@jamstec.go.jp

List of supplemental materials

Supplemental figures

**Fig. S1** Overview of the gravity coring system used during IODP Expedition 386.

**Fig. S2** Principal coordinates analysis of microbial community compositions in bottom water and sediments based on the weighted UniFrac distance matrix.

**Fig. S3** Obtaining single-amplified genomes (SAGs) from Atribacterota JS1 in Japan Trench sediments.

**Fig. S4** Methane concentration and archaeal diversity in the surface sediment of the Japan Trench.

Supplemental table

**Table S1** Sampling locations and holes during IODP Expedition 386.

**Table S2** Summarized geochemical data.

**Table S3** The quality of genomes obtained using single-cell and shotgun metagenomic sequencing in this study.

**Table S4** The genome statistic of JS1 SAGs (≥80% completeness and ≤10% contamination) and MAGs in the Japan Trench (≥50% completeness and ≤10% contamination).

**Table S5** Detection of functional genes in all Atribacterota genome dataset (≥80% completeness and ≤10% contamination).

Supplemental figures


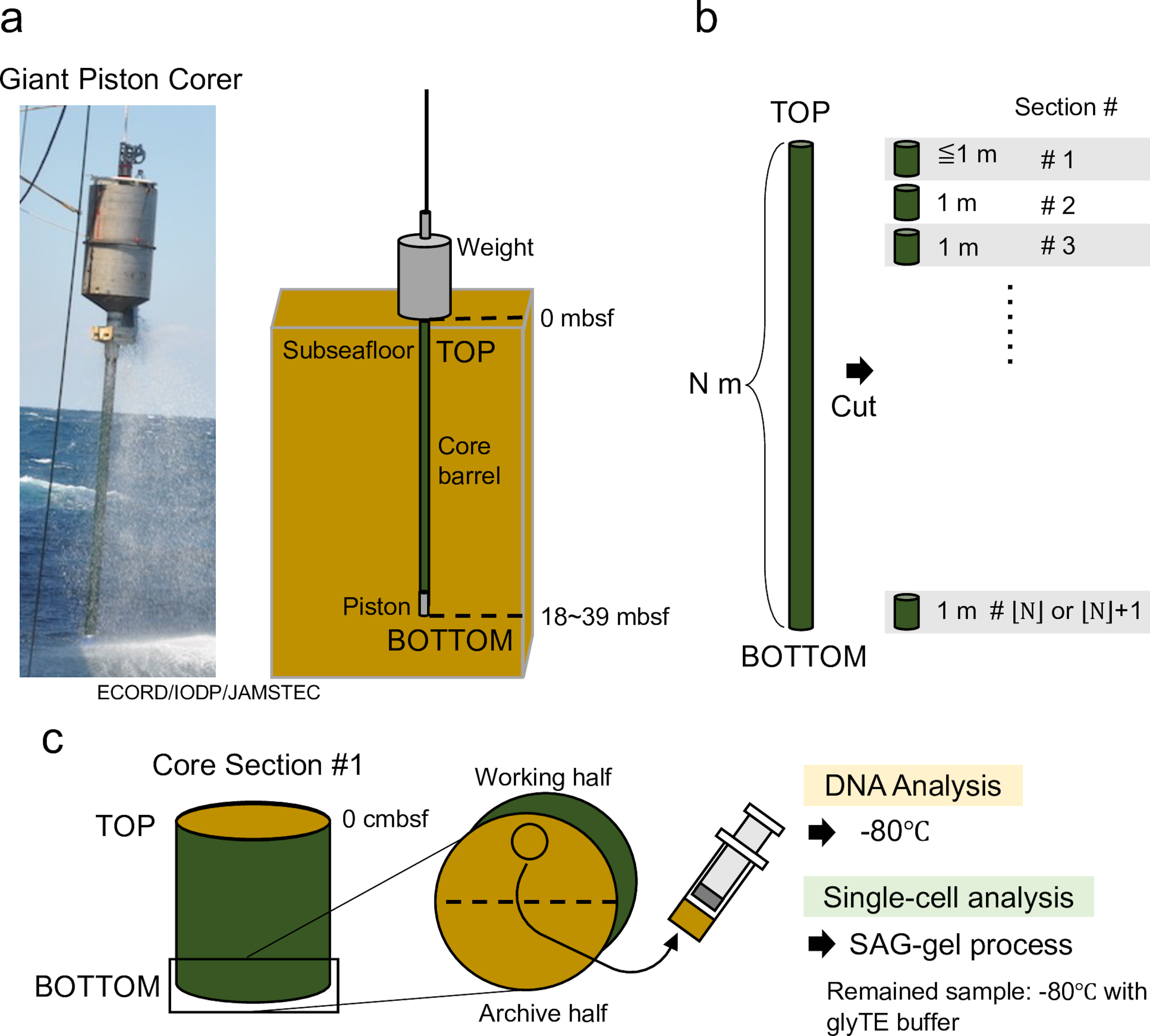


**Fig. S1 Overview of the gravity coring system used during IODP Expedition 386.** (a) An image of the Giant Piston Corer drilling into subseafloor sediments. (b) The process of cutting the GPC core at approximately 1-m interval numbering sections. (c) Sediment sampling for microbial analysis used in this study. The samples were obtained from the bottom end of core section #1 using a tip-cut syringe.


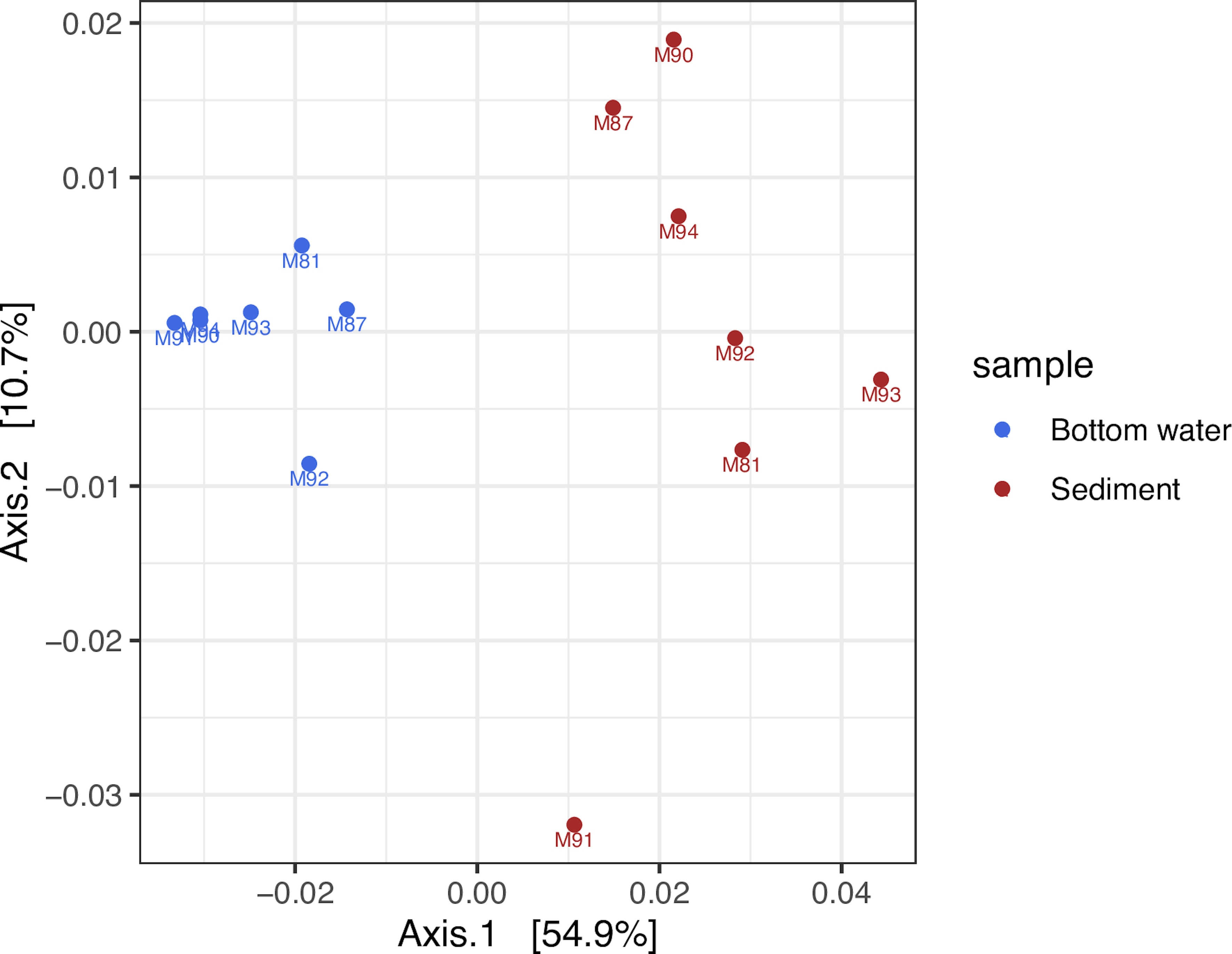


**Fig. S2 Principal coordinates analysis of microbial community compositions in bottom water and sediments based on the weighted UniFrac distance matrix.** The color indicates the sample category, and the text indicates the sampling location.


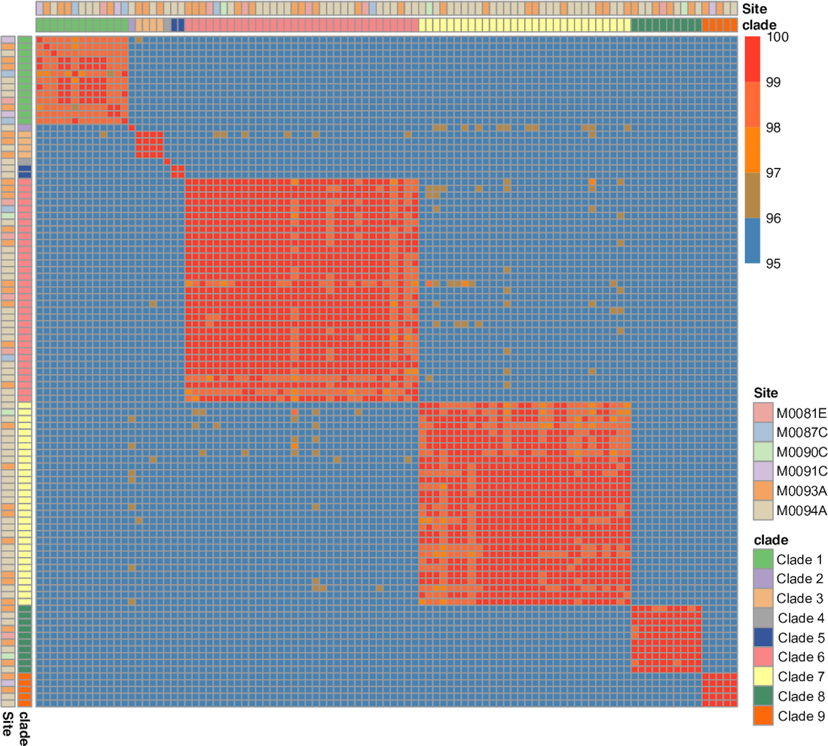


**Fig. S3 Obtaining single-amplified genomes (SAGs) from Atribacterota JS1 in Japan Trench sediments.** The heatmap shows the sequence identity (%) of single-copy marker genes in CheckM. Clustering of SAGs for Atribacterota JS1 based on the similarity of single-copy marker (≥97%) revealed nine distinct JS1 clades in the Japan Trench sediments.


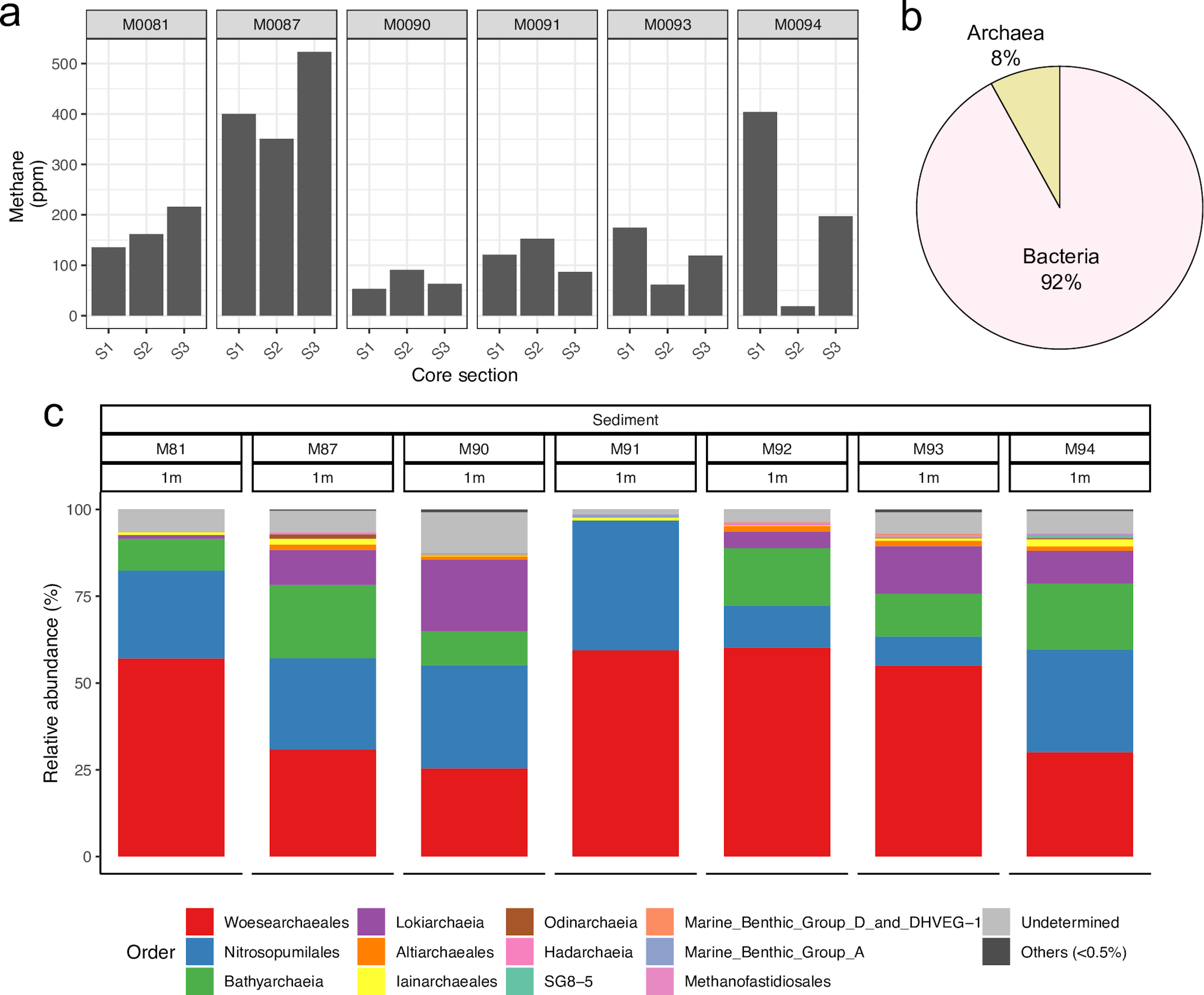


**Fig. S4 Methane concentration and archaeal diversity in the surface sediment of the Japan Trench.** (a) Methane concentration in the sediment of core sections #1–3 (S1, S2, and S3). (b) Proportion of total reads assigned to Archaea, along with percentages of bacterial reads using 515F/806R (V4) universal prokaryotic primers. (c) Archaeal abundance at the order level in the Japan Trench sediments.
